# Declining Death Rates Reflect Progress against Cancer

**DOI:** 10.1371/journal.pone.0009584

**Published:** 2010-03-09

**Authors:** Ahmedin Jemal, Elizabeth Ward, Michael Thun

**Affiliations:** 1 Surveillance and Health Policy Research, American Cancer Society, Atlanta, Georgia, United States of America; 2 Epidemiology Department, American Cancer Society, Atlanta, Georgia, United States of America; London School of Hygiene and Tropical Medicine, Peru

## Abstract

**Background:**

The success of the “war on cancer” initiated in 1971 continues to be debated, with trends in cancer mortality variably presented as evidence of progress or failure. We examined temporal trends in death rates from all-cancer and the 19 most common cancers in the United States from 1970–2006.

**Methodology/Principal Findings:**

We analyzed trends in age-standardized death rates (per 100,000) for all cancers combined, the four most common cancers, and 15 other sites from 1970–2006 in the United States using joinpoint regression model. The age-standardized death rate for all-cancers combined in men increased from 249.3 in 1970 to 279.8 in 1990, and then decreased to 221.1 in 2006, yielding a net decline of 21% and 11% from the 1990 and 1970 rates, respectively. Similarly, the all-cancer death rate in women increased from 163.0 in 1970 to 175.3 in 1991 and then decreased to 153.7 in 2006, a net decline of 12% and 6% from the 1991 and 1970 rates, respectively. These decreases since 1990/91 translate to preventing of 561,400 cancer deaths in men and 205,700 deaths in women. The decrease in death rates from all-cancers involved all ages and racial/ethnic groups. Death rates decreased for 15 of the 19 cancer sites, including the four major cancers, with lung, colorectum and prostate cancers in men and breast and colorectum cancers in women.

**Conclusions/Significance:**

Progress in reducing cancer death rates is evident whether measured against baseline rates in 1970 or in 1990. The downturn in cancer death rates since 1990 result mostly from reductions in tobacco use, increased screening allowing early detection of several cancers, and modest to large improvements in treatment for specific cancers. Continued and increased investment in cancer prevention and control, access to high quality health care, and research could accelerate this progress.

## Introduction

Temporal trends in death rates are the most reliable measure of progress against cancer as they reflect improvements in prevention, early detection and treatment. Although age-standardized cancer death rates in the U.S. have been decreasing since the early 1990s [1], [2], [3], [4], some reviewers cite limited improvement in death rates as evidence that the “war on cancer”, which was initiated in 1971 by President Nixon's administration, has failed [5], [6], [7], [8], [9]. Many of these analyses fail to account for the dominant and dramatic increase in cancer death rates due to tobacco-related cancers in the latter part of the 20^th^ century. Trends in cancer death rates in the U.S. and other industrialized countries provide important insight into the factors associated with economic development and Western lifestyles that drive cancer rates upward as well as the most effective measures to counteract these changes and reduce the cancer burden. In this paper, we examine trends in death rates for all cancers combined and 19 common cancers from 1970–2006 and review the contribution of prevention, early detection, and treatment to reducing cancer death rates. We also provide estimates of the contribution of specific cancer sites to the overall decline in death rates, the number of deaths averted or postponed and the years of potential life gained.

## Methods

We obtained nationwide cancer mortality data for 1970 to 2006 from the SEER*Stat database [10], which defines major cancer sites consistently over time in order to maximize comparability across international classifications of diseases (ICD) versions and facilitate reporting of long term mortality trends [11].

Death rates were directly age-standardized to the 2000 US standard population for all cancers combined and 19 of the most common cancers by using SEER*Stat software [12]. Use of the 2000 US standard population was adopted by Federal and private public health agencies in 2002 to more accurately reflect the contemporary mortality rates, given the aging of the U.S. population [13]. For example, the age-adjusted rates for all cancers combined and for the four major cancer sites in 2000 are 15%–50% higher when adjusted to the 2000 population standard than to the 1970 standard [14].

Temporal trends from 1970 through 2006 for each cancer by sex were described using joinpoint regression analysis (permutation test) [15], which involves fitting a series of joined straight lines (a maximum of four joinpoints) on a log scale to the trends in the annual age-standardized rates [15]. The resultant trends of varying time periods were described by annual percent change (APC), i.e., the slope of the line segment (weighted least square regression, two-sided t test, P<0.05) [15]. Similarly, we examined the trend in death rates with narrower intervals of attained age for all cancers combined (20–49, 50–59, 60–69, 70–79, ≥80) and for the four most common cancers (lung, colorectum, prostate, female breast) (20–49, 50–64, ≥65). We also examined the trend in death rates for all cancers combined and for the four major cancer sites by age and race/ethnicity (whites, blacks, Indians/Alaska Natives [AI/AN], Asian Pacific Islanders [API], Hispanics). For AI/AN, API, and Hispanics, analyses were restricted to data from 1990–2006 because mortality data for these ethnic groups were not uniformly available before 1990. For lung cancer, we examined the 5-year age-specific rates by year of birth beginning at age 30–34.

For cancer sites which had lower death rates in 2006 than in 1990/91 (the years when all-cancer death rates peaked in men and women, respectively), we calculated the contribution of each individual site to the total decrease in the overall cancer death rates. We also estimated the total number of cancer deaths averted due to the decline in the overall age-standardized cancer death rates since the peak, in 1990 for males and 1991 for females, by calculating the number of expected deaths in each calendar year from 1990/91 through 2006 had the death rates not decreased. This was done by multiplying the sex- and age-specific cancer death rates in the peak year to the corresponding sex- and age-specific populations, and then summing the difference between the number of expected and observed deaths in each age group and calendar year for men and for women separately over the 15 or 16 year interval. This method provides the net effect of both decreases and increases death rates for specific cancer sites.

As additional measure for the impact of declining cancer death rates on population health, we calculated years of potential life lost (YPLL) due to cancer before age 75 for 2006 based on the observed cancer deaths in 5-year age interval. We compared this to the YPLL that would have been expected had the 1970 age-specific cancer death rates continued to apply in 2006. YPLL gives more weight to deaths occurring at younger ages [16].

## Results

**Figure 1:**
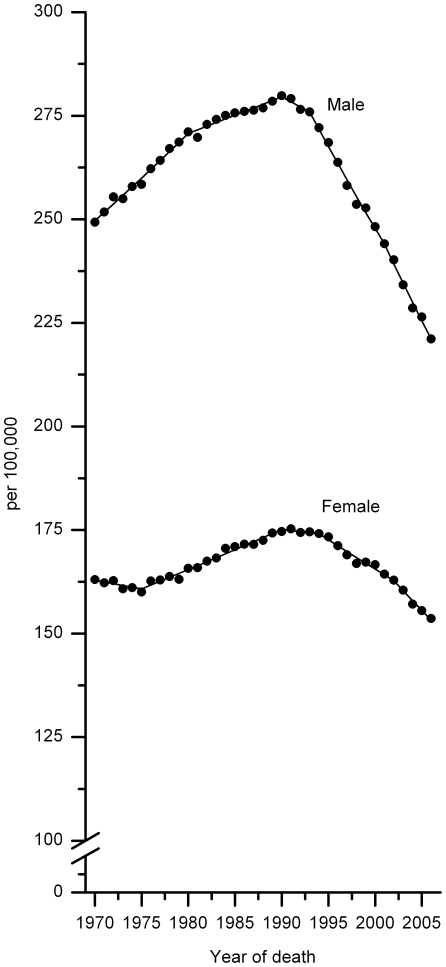
Trends in age-standardized death rates for all cancers combined by sex, 1970–2006. Dots represent observed rates and solid lines fitted rates.

**Figure 2:**
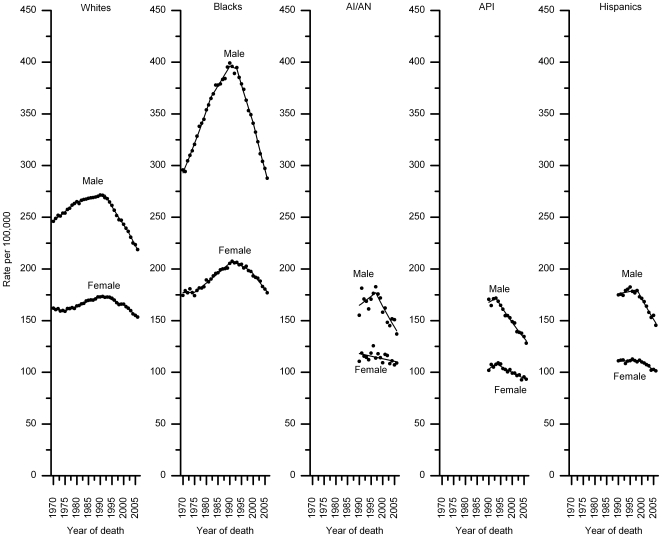
Trends in age-standardized all-cancer death rates by race/ethnicity, 1970–2006. AI/AN: American Indians and Alaska Natives; API: Asian and Pacific Islanders. Dots represent observed rates and solid lines fitted rates.

**Figure 3:**
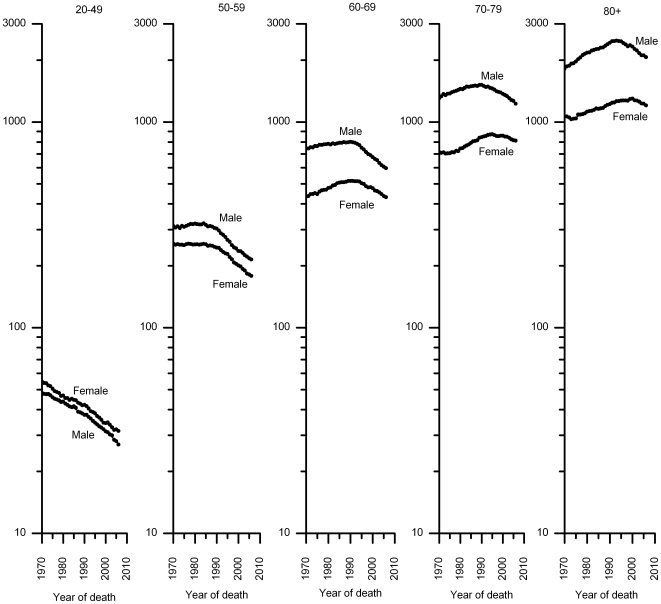
Trends in age-specific death rates (log scale) for all cancers combined, 1970–2006. Dots represent observed rates and solid lines fitted rates.

For all cancers combined, death rates (per 100,000) in men increased from 249.3 in 1970 to 279.8 in 1990, and then decreased to 221.1 in 2006 (Figure 1), yielding a relative decline of 21% and 11% from the 1990 (peak year) and 1970 (baseline) rates, respectively. Similarly, the death rate from all-cancers combined in women increased from 163.0 in 1970 to 175.3 in 1991, and then decreased to 153.7 in 2006, a relative decline of 12% and 6% from the 1991 (peak year) and 1970 rates, respectively. Reductions in age-standardized death rates for all cancers combined since the early 1990 were observed in each major racial and ethnic group, although the onset and magnitude of the decreases vary (Figure 2, Table S1). For example, compared to white women, the decrease in AI/AN women was smaller and the decrease in Hispanic women began later. Notably, black men and women still have 20%–50% excess overall cancer death rates compared to their white counter parts, the group with the second highest mortality rates. During the most recent time period (the last joinpoint interval), rates also decreased in each age group, with the declines beginning progressively earlier and becoming larger, in proportionate terms, with decreasing age (Figure 3, Table S2).

**Figure 4:**
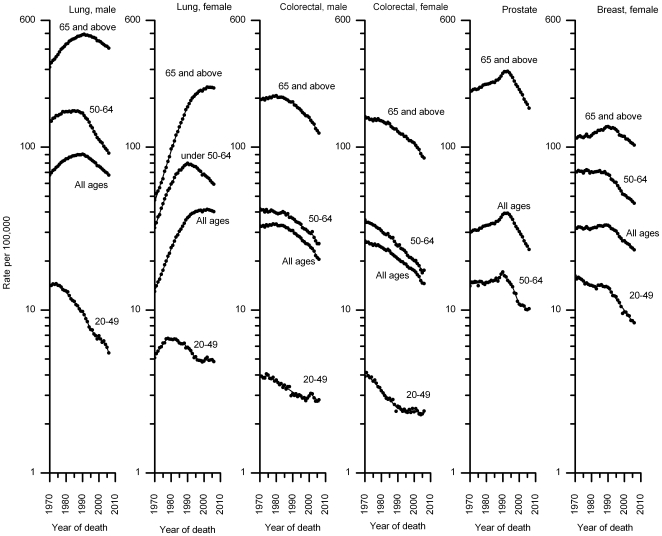
Trends in age-standardized death rates (log scale) for major cancers by age, 1970–2006. Dots represent observed rates and solid lines fitted rates.

Figure 4 shows trends in death rates for all races combined for the four major cancer sites by sex and age. Rates decreased for each major cancer site and age group, although the onset and magnitude of reduction varies by sex and age, especially for lung cancer (Table S3). Among men, lung cancer death rates continued to decrease since 1990 in men, with the decreases beginning earlier in younger ages. Among women, in contrast, the decreases in lung cancer rates at each age were smaller and began much later. The age-specific trends by year of birth show that lung cancer death rates are decreasing among men and women born after the mid 1920s and 1930s, respectively (Figure S1). Similar to the trends for all races combined, mortality trends for the four major cancer sites (except for lung cancer in women) also decreased for each major racial and ethnic group since the early 1990s, although the onset and magnitude of reductions vary (Table S4). For example, the average decrease in colorectal cancer death rates since 1990 was larger in whites than in most of the other racial and ethnic groups.

**Figure 5:**
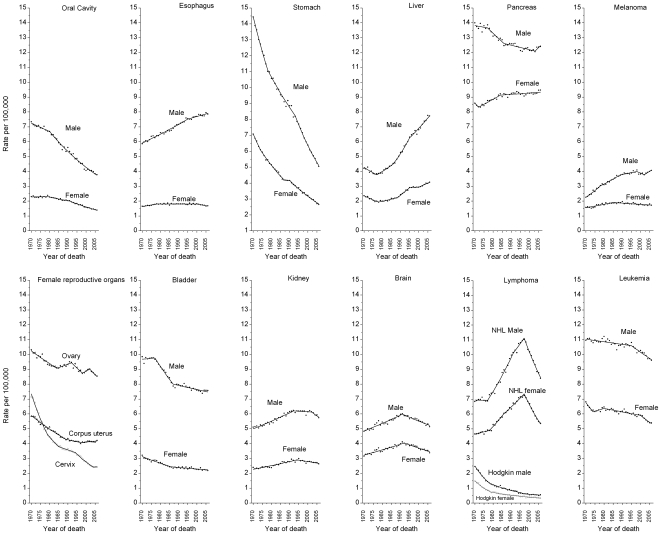
Trends in age-standardized death rates for additional 15 select cancers, 1970–2006. Symbols represent observed rates and solid lines fitted rates.

**Figure 6:**
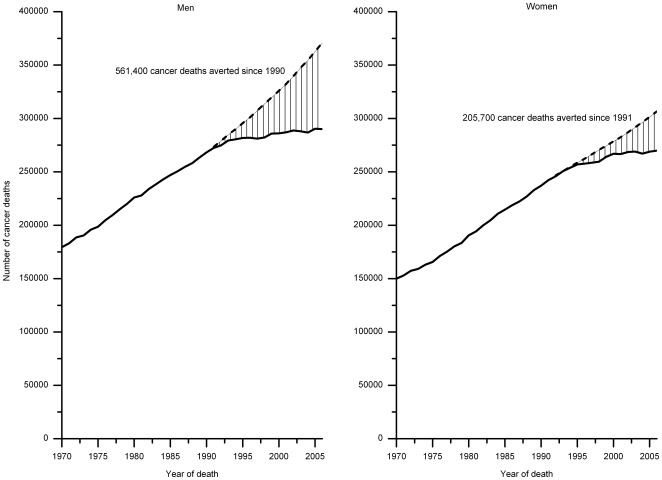
Total number of cancer deaths averted due to reduction in cancer death rates since 1990/1991. Solid lines represent observed number of cancer deaths and dashes expected cancer deaths. Expected cancer deaths were based on had the peak rates in men (1990) and women (1991) prevailed through 2006.

Figure 5 shows trends in age-standardized death rates from 1970–2006 for 15 additional cancer sites by sex, with the APC estimates given in Table S5. During the most recent time period, death rates decreased for cancers of the oral cavity, stomach, bladder, kidney, brain, and Non-Hodgkin lymphoma, and leukemia in both males and females and for cancers of the esophagus and ovary and melanoma and Hodgkin lymphoma in females. In contrast, rates increased for esophagus cancer and melanoma in men, liver cancer in both men and women, and pancreas cancer in women. Death rates stabilized for pancreatic cancer and Hodgkin lymphoma in men and for cervix and corpus and uterus cancers in women. Notably, the 2006 death rates for Hodgkin lymphoma in men, cervical cancer in women, and stomach cancer in both men and women were less than one-third of the 1970 rates. For the sites which increased during this time interval, death rates nearly doubled for melanoma and liver cancer in men and tripled for lung cancer in women. The downturn in lung cancer death rates among women did not occur until 2002, reversing the continuously increasing trend observed since national compilation of vital statistics began in 1930s. It is noteworthy that the largest absolute decreases in death rates (per 100,000) that occurred between 1990/91 and 2006 were observed for lung cancer in men (23.2) and breast cancer in women (9.3). These accounted for 37% of the total decrease in all-cancer death rates in men and women during this time period (Table 1). The decrease in prostate cancer death rates accounted for 24% of the total decrease in men; reduction in colorectal cancer death rates accounted for 17% and 23% of the total decrease in men and women, respectively. Overall, the continued decrease in all-cancer death rates from 1990–2006 in men and 1991–2006 in women translates to averting of 561,400 cancer deaths in men and 205,700 cancer deaths in women that would have occurred if the 1990/91 rates were to prevail afterwards (Figure 6).

**Table 1:** The contribution of individual cancer sites to the decrease in all-cancer death rates, 1990–2006.

	Death Rate	Death Rate			
	per 100,000	per 100,000	Change	Change	% Contribution†
	1990*	2006	Absolute	%	
**Male**					
All Malignant Cancers	279.8	221.1	−58.7	−21.0	
***Decreasing***					
Lung & Bronchus	90.6	67.5	−23.1	−25.5	37.2
Prostate	38.6	23.6	−15.0	−38.9	24.2
Colorectum	30.8	20.5	−10.3	−33.4	16.5
Stomach	8.9	5.0	−3.8	−43.1	6.1
Oral Cavity & Pharynx	5.6	3.8	−1.8	−32.6	2.9
Non-Hodgkin Lymphoma	10.0	8.4	−1.6	−15.6	2.5
Leukemia	10.7	9.6	−1.1	−10.3	1.8
Brain & other nervous system	6.0	5.1	−0.8	−14.1	1.4
Larynx	3.0	2.2	−0.8	−26.2	1.3
Myeloma	4.8	4.4	−0.5	−9.7	0.8
Kidney & renal pelvis	6.2	5.7	−0.4	−6.9	0.7
Urinary bladder	8.0	7.6	−0.4	−5.0	0.6
Hodgkin Lymphoma	0.9	0.6	−0.3	−34.7	0.5
Other	39.8	37.6	−2.2	−5.6	3.6
Total	263.6	201.5	−62.1		100.0
***Increasing***					
Esophagus	7.2	7.9	0.7	9.7	
Liver & Intrahepatic Bile duct	5.3	7.7	2.5	46.5	
Melanoma of the Skin	3.8	4.1	0.3	7.1	
Total	16.2	19.7	3.4		

	Death Rate	Death Rate			
	per 100,000	per 100,000	Change	Change	% Contribution†
	1991*	2006	Absolute	%	
**Female**					
All Malignant Cancers	175.3	153.7	−21.6	−12.3	
***Decreasing***					
Breast	32.7	23.4	−9.2	−28.3	36.7
Colorectum	20.3	14.5	−5.8	−28.4	22.9
Non-Hodgkin Lymphoma	6.7	5.4	−1.4	−20.3	5.4
Stomach	4.0	2.6	−1.4	−34.0	5.4
Cervix Uteri	3.5	2.4	−1.1	−30.7	4.3
Ovary	9.5	8.5	−1.0	−10.2	3.9
Leukemia	6.3	5.4	−0.9	−14.6	3.7
Brain & other nervous system	4.1	3.4	−0.7	−17.6	2.9
Oral Cavity & Pharynx	2.0	1.4	−0.6	−31.6	2.5
Other	36.7	33.6	−3.1	−8.4	12.3
Total	125.9	100.7	−25.2	−20.0	100.0
***Increasing***					
Lung & Bronchus	37.6	40.2	2.6	6.8	
Liver & Intrahepatic Bile duct	2.5	3.3	0.8	30.0	
Pancreas	9.3	9.5	0.2	2.1	
Total	49.4	52.9	3.5		

*Death rates from all cancer peaked in 1990 for men and 1991 for women.

†This calculation is based on each cancer site's contribution to the increasing or decreasing portion of the total cancer death rate, depending on the individual site's trend; it does not represent the contribution to the net decrease in cancer death rates.

Cancer deaths in 2006 resulted in 4.4 million YPLL among persons under age 75. If the 1970 age-specific death rates persisted through 2006, the YPLL in 2006 would have been 6.4 million. Thus, the decrease in cancer death rates in younger ages during this 36 years time interval resulted in about 2.0 million years of potential life gained under age 75 in the year 2006.

## Discussion

Contrary to the pessimistic news from the popular media, overall cancer death rates have decreased substantially in both men and women whether measured against baseline rates in 1970/71 when the National Cancer Act was signed by President Nixon or when measured against the peak rates in 1990/91. The decrease involves all age groups, all major racial and ethnic groups, and 15 of the 19 cancer sites considered in this analysis. Reductions in death rates from the four major cancer sites (lung, female breast, prostate, colorectum) accounted for about 60%–80% of the total decrease in all-cancer death rates since 1990/91. The decrease in all-cancer death rates since the peak years of 1990/91 translates to averting 561,400 cancer deaths in men and 205,700 cancer deaths in women. Further, the decrease in death rates at younger ages since 1970 resulted in about 2.0 million years of potential life gained under age 75 in 2006 alone.

Advances in prevention, early detection, and treatment all have contributed to this progress in reducing death rates from cancer [2], [17], [18], [19], [20]. The decreases in death rates from lung cancer and other smoking-related cancers resulted mostly from reductions in smoking prevalence since the 1950s [2], [21]. Most of the reduction in lung cancer death rates observed since in 1990 in men and since in 2002 in women represents smoking cessation that began among educated men and women in 1950s; the full benefits of reduced initiation among adolescents will emerge in the future as these generations age. Between 1965 and 2006, current smoking prevalence among all U.S. adults 18 year and above decreased by 54% (from 51% to 24%) in men and by 46% (from 34% to 18%) in women [22]. Despite these substantial reductions in smoking prevalence and mortality from smoking-related cancers, cigarette smoking still accounts for approximately 30% of all cancer deaths, with lung cancer contributing most (80%) of these deaths [23]. About 45 million adults continue to smoke cigarettes. Decreasing initiation and increasing cessation through proven tobacco control interventions continue to be important priorities for reducing cancer mortality in the short and long term.

The dramatic decrease in stomach cancer death rates that has occurred in most economically developed or transitioning countries is thought to result from the combination of reduced prevalence of *Helicobacter Pylori* infection and the benefits of refrigeration [24], [25]. The latter has reduced reliance on smoked and salted preserved foods and increased the availability of fresh fruits and vegetables. Since the early 1930s when national mortality records became available, stomach cancer dropped from the leading cause of cancer death to 11^th^ in men and from 2^nd^ to 12^th^ in women [26]. The decreases in mortality from female breast cancer since 1989 and from colorectal cancer since the 1980s largely reflect a combination of earlier diagnosis through screening and improved treatment [17], [19], [27]. In the case of colorectal cancer, screening decreases incidence as well as mortality rates through the detection and removal of precancerous polyps [28]. Similarly, the large decrease in cervical cancer death rates over the past 40–50 years resulted mostly from removal of precancerous lesions through Pap smear screening beginning in 1950s [29], [30]. However, rates have flattened in the three most recent data years (2004–06), and it requires 2 or more additional data years to ascertain this new pattern. Screening for breast cancer for women beginning at age 40 and for colorectal cancer for adults beginning at age 50 were introduced in early 1980s, although national monitoring for these data did not begin until 1987 [31]. According to the most recent data from the National Health Interview Survey, the percentage of women 50 or above who had mammography in the past two years increased from 29% in 1987 to 70% in 2000, and then slightly decreased to 67% in 2005 [32], [33]. During the same time period, the percentage of adults 50 or above who had screening for colorectal cancer according to guideline increased from 27% to 47% [33], [34].

The substantial decrease in prostate cancer death rates since the mid 1990s thought to reflect improved treatment and the introduction and wide dissemination of Prostate Specific Antigen (PSA) blood test in men 50 or above since the late 1990s [20], [35], [36], [37]. However, a recent randomized trial in the U.S. failed to demonstrate the benefit for PSA testing in reducing deaths from prostate cancer, although another trail in Europe showed a benefit [38], [39]. Further, prostate cancer death rates have decreased substantially in many other western countries where PSA testing is not widely practiced [40], [41], [42]. The increase in prostate cancer death rates in late in 1980 in the U.S. thought to reflect attribution bias [43]. The introduction and dissemination of new treatments for Hodgkin lymphoma, leukemia, and testicular cancer between 1950 and 1970 and for non-Hodgkin lymphoma in the mid 1990s contributed to the rapid reductions in mortality from these diseases [44], [45], [46], [47], [48].

In contrast to the long-term decreases in death rates for several cancer sites, rates increased for cancers of the liver (both sexes), esophagus (men), pancreas (women), and melanoma (men). The increase in liver cancer is generally attributed to the increasing prevalence of Hepatitis C infections [49], but may also be influenced by rising obesity rates [50]. Both tobacco use and obesity are thought to contribute to the long-term increase in mortality from pancreas and esophagus cancers, but only the obesity epidemic could contribute to the recent increases [25], [51], [52], [53]. Obesity increases gastroesophageal reflux disease, the major risk factor for adenocarcinoma of the esophagus [54], [55]; adenocarcinoma of the esophagus has been increasing for decades [56]. The increase in melanoma death rates in men is generally attributed to historical birth cohort trends in sun exposure [57].

The greater decrease in the overall cancer death rates in men than women largely due to differences in mortality trends from lung cancer which accounts for about 80% of all smoking attributable cancer deaths and nearly 30% of the total cancer deaths in the U.S. [21], [58], [59]. Lung cancer death rates have continued to decrease since 1990 in men, while they continued to increase through the mid 2000 in women. Trends in lung cancer also explain, in part, why the decrease in overall cancer deaths rates began earlier and were larger in proportionate terms in the younger than in the older age groups. Smoking prevalence and lung cancer peaked in those men born around 1920s and in women born around the late 1930s, now in their 70–90s. Younger cancer patients are also more likely to receive aggressive treatment and to participate in clinical trials [17].

Racial/ethnic disparities in the reduction of overall or cancer-specific death rates during the most recent time period reflect differences in historical exposures to risk factors/and or access to health care [4], [19], [60]. For example, the average decrease in colorectal cancer death rates since 1990 appear to be larger among whites compared to most of the other racial/ethnic group in part because whites have the highest colorectal screening rates of all racial/ethnic groups [33], and white colorectal cancer patients are more likely to receive the standard treatment [17], [61].

Similar trends in death rates for most of the cancer sites described here have been reported in several western countries. For instance, lung cancer mortality rates among men continued to decrease since at least the early 1990s in Several European countries, Australia, and Canada [62], [63], [64] because as in the U.S. the tobacco epidemic in men in these countries occurred before the middle of the 20^th^ century [65]. Over the past 20 years, breast cancer mortality trends have decreased in several Western countries because of early detection through mammography, improved treatment, and reduced use of post menopausal hormone [66], [67], [68]. Remarkable decreases in cervical cancer death rates have also been noted in several countries with long-established cervical cancer screening programs, including European countries, Canada, and Australia [69], [70], [71].

The value of measuring temporal trends in death rates rather than in relative survival is that death rates are much less susceptible to artifactual changes from screening and lead time or length bias. However, mortality trends may be affected by changes in the quality of death certificates [72], [73] and changes in classification of causes of death [74]. Although the quality of cause of death certification may have improved over the years, this is unlikely to account for the large drops in mortality for either specific cancer sites or all-cancers combined. No significant discontinuities in the mortality trends occurred with the implementation of ICD-9 or ICD-10 for any of the cancer sites considered in this analysis [75]. Further, any effect of the introduction of revised ICD-coding on mortality trend is transient, and should not affect long-term trends [74]. Another limitation of our analysis is we presented data for broad racial and ethnic groups only, and this may mask important differences in cancer rates and trends by country of origin within each racial/ethnic group. Among API women for example, cervical cancer rate are almost three times as high in Vietnamese as in Chinese or Japanese in part because Vietnamese women are recent immigrants, are poorer, and have less access to cervical cancer screening [76].

In addition to the steady reduction in mortality trends, basic and clinical research has also contributed to better quality of life for cancer survivors. These include relief from pain and other distressing symptoms, sentinel lymph node biopsy for removal of fewer regional lymph nodes, breast conserving surgery for patients with breast cancer, limb sparing surgery for patients with sarcoma, sphincter sparing surgery for patients with rectal cancer, and voice preservation for patients with squamous cell of the head and neck cancers. Further, both chemo and radiation treatments have been improved so that cancer cells are better targeted and adverse health effects of healthy cells are minimized.

In summary, progress in reducing cancer death rates is evident whether measured against baseline rates in 1970 or in 1990. Downturns in overall cancer death rates since the early 1990s are largely a result of tobacco control efforts beginning in the 1960s, screening and early detection for several cancers disseminated in the 1980s and 1990s, and modest to large improvements in treatment and survival for specific cancers. Continued and increased investment in cancer prevention and control programs, access to high quality health care, and basic and clinical research could accelerate this progress.

## Supporting Information

Figure S1 Trends in age-specific lung cancer* death rates (log scale) by year of birth, United States, 1865–1970. Dots represent observed rates and solid lines fitted rates. *Includes lung, bronchus, pleura, and trachea. The points vertically above each cohort year portray the cohort's age-specific mortality experience.
(0.80 MB TIF)

Table S1 Trends in Overall Mortality for All Cancers by Race and Sex, 1970–2006 and 1990–2006.
(0.04 MB XLS)

Table S2 Trends in All Cancer Mortality by Age and Sex, 1970–2006.
(0.03 MB XLS)

Table S3 Trends in Mortality for Common Cancers by Sex and Age, 1970–2006.
(0.03 MB XLS)

Table S4 Trends in Mortality for Common Cancers by Sex and Race, 1990–2006.
(0.03 MB XLS)

Table S5 Trends in Mortality for Additional Cancers by Sex, 1970–2006.
(0.03 MB XLS)
